# Role of IL-1β in Experimental Cystic Fibrosis upon *P. aeruginosa* Infection

**DOI:** 10.1371/journal.pone.0114884

**Published:** 2014-12-12

**Authors:** Jennifer Palomo, Tiffany Marchiol, Julie Piotet, Louis Fauconnier, Marieke Robinet, Flora Reverchon, Marc Le Bert, Dieudonnée Togbe, Ruvalic Buijs-Offerman, Marta Stolarczyk, Valérie F. J. Quesniaux, Bob J. Scholte, Bernhard Ryffel

**Affiliations:** 1 CNRS, UMR7355, Orleans, France; 2 Experimental and Molecular Immunology and Neurogenetics, University of Orléans, Orléans, France; 3 Artimmune, Orléans, France; 4 Erasmus MC, Cell Biology department, Rotterdam, The Netherlands; 5 Institute of Infectious Disease and Molecular Medicine, IDM, Cape Town, South Africa; University of North Dakota, United States of America

## Abstract

Cystic fibrosis is associated with increased inflammatory responses to pathogen challenge. Here we revisited the role of IL-1β in lung pathology using the experimental F508del-CFTR murine model on C57BL/6 genetic background (*Cftr* ^tm1eur^ or d/d), on double deficient for d/d and type 1 interleukin-1 receptor (d/d X IL-1R1^−/−^), and antibody neutralization. At steady state, young adult d/d mice did not show any signs of spontaneous lung inflammation. However, IL-1R1 deficiency conferred partial protection to repeated *P. aeruginosa* endotoxins/LPS lung instillation in d/d mice, as 50% of d/d mice succumbed to inflammation, whereas all d/d x IL-1R1^−/−^ double mutants survived with lower initial weight loss and less pulmonary collagen and mucus production, suggesting that the absence of IL-1R1 signaling is protective in d/d mice in LPS-induced lung damage. Using *P. aeruginosa* acute lung infection we found heightened neutrophil recruitment in d/d mice with higher epithelial damage, increased bacterial load in BALF, and augmented IL-1β and TNF-α in parenchyma as compared to WT mice. Thus, F508del-CFTR mice show enhanced IL-1β signaling in response to *P. aeruginosa*. IL-1β antibody neutralization had no effect on lung homeostasis in either d/d or WT mice, however *P. aeruginosa* induced lung inflammation and bacterial load were diminished by IL-1β antibody neutralization. In conclusion, enhanced susceptibility to *P. aeruginosa* in d/d mice correlates with an excessive inflammation and with increased IL-1β production and reduced bacterial clearance. Further, we show that neutralization of IL-1β in d/d mice through the double mutation d/d x IL-1R1^−/−^ and in WT via antibody neutralization attenuates inflammation. This supports the notion that intervention in the IL-1R1/IL-1β pathway may be detrimental in CF patients.

## Introduction

Cystic fibrosis (CF) is an autosomal recessive genetic disease that affects 1 newborn out of 3,500 in the USA (WHO), and 1 per 2,000–3,000 in Europe, with an average 40-year life expectancy. This pathology is caused by mutations within the gene encoding the CFTR (*Cystic Fibrosis Transmembrane Conductance Regulator*) chloride channel [1]. In humans, the most common mutation (F508del) found in patients is a deletion of phenylalanine 508 (F508del) in the CFTR chloride channel [2], the modified protein is not efficiently folded and is rapidly degraded [3]. CF affects secretory epithelia from different organs, leading to gastro-intestinal tract pathology, with a reduced pancreatic digestive enzyme production [4] and chronic intestinal malabsorption [5]. CF mortality and morbidity are mainly due to respiratory disease, characterized by the production of abnormally viscous mucus, plugging of distal airways, and increased susceptibility to chronic infections with opportunistic bacteria, excessive lung inflammation, bronchiectasis and fibrosis leading to progressive loss of lung function [6]. The exact molecular link between CFTR mutations and hypersensitivity to infections remains unclear and controversial. In CF patients, *S. aureus* predominates in the lung of children and teenagers, while *P. aeruginosa* prevails in adults [7]. Exopolysaccharide-enriched biofilms produced by *P. aeruginosa* increase the mucus viscosity, resistance to antibiotics and host immune effectors. Chronic bacterial infections are common in CF patients and facilitates lung inflammation, mucous obstruction and tissue remodeling, resulting in fatal loss of function [1]. CF lungs display excessive inflammatory response, especially with increased neutrophil recruitment [8], the mechanism of this phenomenon is not adequately explained. However, intervention in this process likely will benefit CF patients.

The role of the pro-inflammatory signaling cytokine Interleukin 1β (IL-1β) in CF lung disease has been reported before. *P. aeruginosa* induces IL-1β or IL-18 production through NLRC4 inflammasome activation [9], [10]. *P. aeruginosa* flagellin and highly acylated LPS is recognized by TLR5 [11] and TLR4 [12] respectively. Human polymorphisms observed in the *IL1B* gene were associated with CF disease [13]. CFTR deficient mice were found to be more susceptible to acute [14], [15] and chronic [16] *P. aeruginosa* infection and display an exacerbated inflammatory response to LPS and *P. aeruginosa* activated alveolar macrophages from F508del mutant mice have enhanced expression of IL-1β [17], [18]. Huaux et al recently showed a deregulated inflammatory and fibrotic response in F508del mutant mice to bleomycin, which is IL-1R1 signaling dependent [19], [20].

Here we revisited the role of IL-1β in the resolution of *P. aeruginosa* infection, in a murine model based on mice carrying the most common CF mutation F508del CFTR [21]–[23]. In this study, we show that excessive activation of IL-1β correlates with increased bacterial load, inflammation and lung damage in F508del CFTR mice. Further, we show that IL-1β antibody neutralization attenuates the inflammatory response to *P. aeruginosa* infection.

## Materials and Methods

### Mice

Mice were on C57BL/6(J) background, wild type (WT), or homozygotes for F508del CFTR mutation in the murine *Cftr* gene (*Cftr* ^tm1eur^ or d/d) [21]–[23], and deficient for type 1 interleukin 1 receptor (d/d X IL-1R1^−/−^) [24]. Mice, obtained from Erasmus MC Rotterdam [21] were bred at TAAM – UPS 44, Orléans (Institut Transgenose) and manipulated in an SPF zone. This study was carried out in strict accordance with the recommendations of CNRS, and in the Guide "Animaleries de laboratoire”. The protocol was approved by the Committee on the Ethics of CNRS of Orléans (Permit Number: CLE CCO 2012-042). All animals are under daily inspection by the animal facility staff and the experimenters. All efforts were made to minimize suffering.

### IL-1β antibody administration

Mice were treated intraperitoneally, once a week for 8 weeks, with anti-IL-1β antibody (Dr. Hermann Gram, Novartis Pharma, Basel) [25], 10 mg/kg. Control mice received 150 µL PBS. Mice were euthanatized 1 week after the last treatment, by carbon dioxide inhalation (80–90% in a dedicated inhalation chamber).

### Repeated LPS induced lung inflammation (4 weeks)

Mice were treated intranasally with 80 µg of *P. aeruginosa* endotoxins/LPS [Sigma Chemical Co., St. Louis, MO] in 40 µL PBS, under isoflurane anesthesia, once a week for 4 weeks. Control mice were untreated. Mice were euthanatized 24 h after the last challenge, by carbon dioxide inhalation (80–90% in a dedicated inhalation chamber). All animals are under daily inspection by the experimenters. Humane endpoints were used during this survival study: in case that mice unexpectedly present obvious health problems (over 20% weight loss, signs of suffering, difficulties in their movements and uptake of water and food, apathy) were sacrificed, by carbon dioxide inhalation (80–90% in a dedicated inhalation chamber).

### *Pseudomonas aeruginosa* infection

Mice were infected with freshly prepared inoculum of *P. aeruginosa* strain 2310.55 of serotype IATS O11. An overnight culture in 10 mL BHI medium was prepared, starting from the frozen stock at 37°C and shaken at 150 rpm. Of this culture, 2.5 mL was taken to start a fresh 10 mL BHI culture. The culture was stopped when an OD of about 0.4 was reached (corresponding to a bacterial titer of about 2×10^8^ bacteria/ml). Mice were anaesthetized with a low dose of intravenous ketamine/xylazine (1.25 mg/ml/0.5 mg/mL) and 40 µl of the bacterial solution or the corresponding vehicle solution (isotonic saline) was applied intranasally using an ultrafine pipette tip. Mice were monitored until they woke up and evaluated 6 h and 24 h after infection.

### IL-1β antibody administration to infected mice

Mice were treated intraperitoneally, with anti-IL-1β antibody (Novartis Pharma, Basel) [25], 200 µg/mice, 15 h and 1 h before *P. aeruginosa* infection. Control mice received 150 µL PBS. Mice were euthanatized 20 h after infection, by carbon dioxide inhalation (80–90% in a dedicated inhalation chamber).

### Bacterial load in lung

Lung total weights were recorded after sacrifice and expressed as a percentage of the body weight. Lung homogenates were prepared in 1.5 ml of isotonic saline solution using a Dispomix tissue homogenizer (Medic Tools). Tenfold serial dilutions of homogenate were plated on BHI agar (BHI 37g/l, Agar 15g/l) plates (Biovalley). Plates were incubated at 37°C and 5% CO_2_ and the numbers of CFU were enumerated after 24 h.

### Bronchoalveolar lavage (BAL)

After CO_2_ inhalation deep euthanasia, bronchoalveolar lavage fluid (BALF) was collected by cannulating the trachea and washing the lung with 1 mL saline at room temperature. The lavage fluid was centrifuged at 2,000 rpm for 10 min at 4°C and the supernatant was stored at −80°C for analysis. The cell pellet was resuspended in PBS, counted in a haemocytometer chamber and cytospin preparations were made using a Shandon cytocentrifuge (1000 rpm for 10 min). The cells were stained with Diff-Quick (Dade Behring, Marburg, Germany) and counted for neutrophils, macrophages, lymphocytes and eosinophils.

### Total RNA extraction and RT-q-PCR from lung

Total mRNA was isolated from homogenized lung using TRI-Reagent (Sigma), purified by RNeasy Mini Kit (Qiagen, Valencia, CA), and quantified by NanoDrop (Nd-1000). Reverse transcription was performed in with SuperScriptIII Kit according manufacturer's instructions (Invitrogen). cDNA was subjected to quantitative real-time PCR using primers for *Il1b, Il6* or *Ccl2* (Qiagen) and GoTaq qPCR-Master Mix (Promega). *GAPDH* and *18S* expression was used for normalization. Raw data were analyzed using the Relative Expression Software Tool (REST, http://www.rest.de.com/).

### Cytokine and chemokine measurement

TNF-α, IL-6, IL-1β and keratinocyte-derived chemokine (KC or CXCL-1) concentrations in BALF and lung homogenates were measured by ELISA (Duoset Kit; R&D Systems) according to the manufacturer's instructions.

### MPO activity in lung

Lungs were homogenized in Dispomix [MedicTools. Zug, CH] with 1.5 mL NaCl 0.9%, and centrifuged. The supernatant was discarded. The pellet was homogenized in 1 mL PBS - HTAB 0.5% - EDTA 1 mM. After centrifugation, 20 µL supernatant were added with: 1 mL HBSS, 200 µL of PBS - HTAB 0.5% - EDTA 1 mM buffer, 100 µL o-Dianisidine (1.25 mg/mL) and 100 µL H_2_O_2_ 0.048%. The reaction was stopped with 100 µL NaN_3_ 1%. MPO (myeloperoxidase) activity was determined as absorbance at 460 nm against medium.

### Protein assay

Total protein concentration was analyzed in BALF, using Bradford assay with BIORAD DC Protein assay kit (Biorad), according to the manufacturer's instructions.

### Histology

Lungs were fixed in 4% buffered formalin, dehydrated in ethanol and embedded in paraffin. Sections (3 µm) were stained with haematoxylin and eosin (H&E), CAB (collagen staining) or PAS (mucus staining) and all lung sections were evaluated by two independent observers for pathological changes, collagen deposition, mucus production and cellular recruitment.

### Statistical analysis

Statistical evaluation of differences between the experimental groups was determined by using Mann-Whitney non-parametric test, or Log Rank test for survival. All tests were performed with GraphPad Prism [GraphPad Software Inc., San Diego, CA, USA; www.graphpad.com]. A *P*-value <0.05 was considered significant and symbolized with *; ** for p<0.01 and *** for p<0.001. A *P*-value>0.05 was considered not significant and symbolized with ns.

## Results

### 1. IL-1β signaling enhances lung inflammation in F508del CFTR mutants in response to LPS

**Figure 1:**
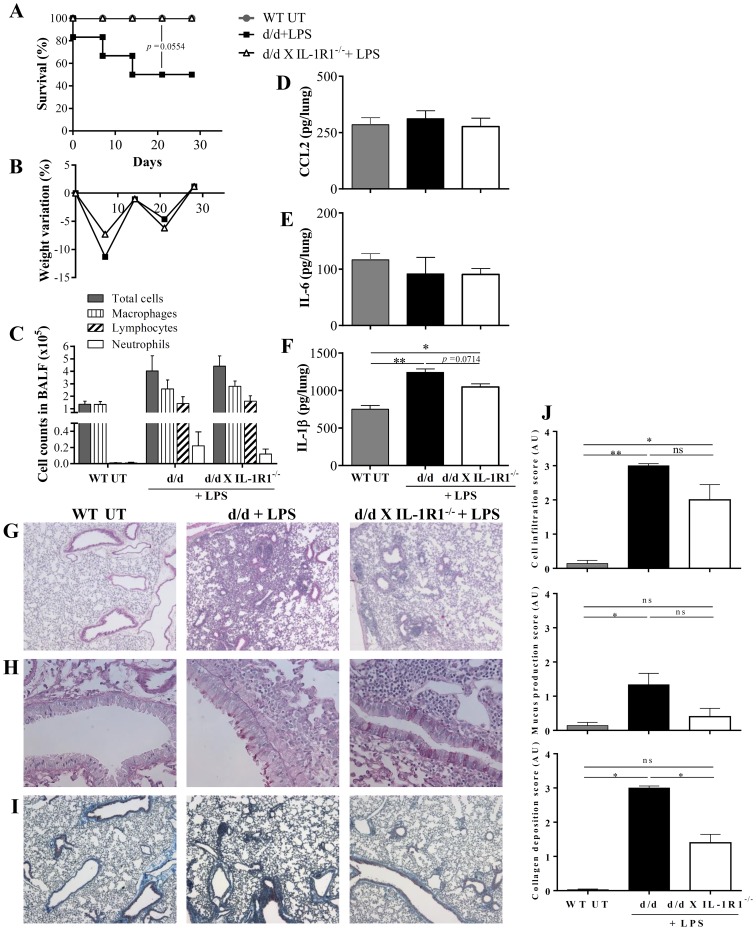
IL-1β participates in pathologic inflammation in F508del CFTR mutants, in response to *P. aeruginosa* LPS. d/d and double KO d/d x IL-1R1^−/−^ mice were treated intranasally with 80 µg of *P. aeruginosa* endotoxins/LPS in 40 µL PBS, once a week for 5 weeks. Untreated WT mice (UT) were used as control. Survival is presented in (A) and body weight variation 24 h after the last LPS challenge in (B). Absolute numbers of cells, (macrophages, lymphocytes and neutrophils) were measured in BALF 24 h after the last LPS challenge (C). CCL2 (D), IL-6 (E) and IL-1β (F) were measured in lung homogenate. Cell infiltration was observed on H&E staining (G), mucus production on PAS staining (H) and collagen deposition on CAB staining (I). Histopathological scores are shown in (J). n = 6–7 initially, n = 3 for d/d+LPS mice 24 h after last challenge instillation. Values are in mean +/− SEM; * for p<0.05 and ns for non-significant.

Since LPS, *P. aeruginosa* [14]–[16] and bleomycin induced injury and inflammation [26] are increased in F508del CFTR (d/d mice), and since the IL-1R1 pathway is involved [20], [27] and required for the development of bleomycin-induced fibrosis [20], we studied the role of the IL-1β pathway in the response to inflammation and lung damage in F508del CFTR mice. In order to induce chronic lung damage, repeated *P. aeruginosa* LPS challenges were performed in d/d and in double mutant d/d x IL-1R1^−/−^ mice, and compared to untreated WT littermate controls. Lung analysis was performed at day 29, 24 h after the last LPS challenge. Survival upon repeated LPS challenge was reduced to 50% in the d/d mice, while 100% of double KO d/d x IL-1R1^−/−^ mice survived (**Fig. 1.A**). The results suggest that in F508del CFTR mutant, the active IL-1β pathway is deleterious in response to repeated inflammatory injury. Reduced survival of d/d mice was apparently associated with higher loss of body weight at day 7, as compared to d/d x IL-1R1^−/−^ mice (**Fig. 1.B**). Nevertheless, all surviving mice recovered their initial weight after 4 weeks, indicating tolerance to repeated LPS challenges. LPS-induced inflammation was confirmed by increased total cell number in BALF (macrophages and lymphocytes) 24 h after the last challenge but no significant difference was observed between d/d and d/d X IL-1R1^−/−^ mice at this time point (**Fig. 1.C**). Similarly, *P. aeruginosa* LPS also induced a recruitment of neutrophils in BALF of both d/d and d/d X IL-1R1^−/−^ mice (**Fig. 1.C**). No increase compared to untreated WT mice of the CCL2 chemokine and IL-6 could be detected in lung homogenate after repeated LPS challenges (**Fig. 1.D** and **E**). These results also suggest tolerance since LPS is known to induce these cytokines after single challenge [28]. Single acute LPS challenge is also known to induce IL-1β [29]. Indeed, the pulmonary IL-1β production was still augmented after repeated LPS challenges in d/d mice, but to a lesser extend in d/d x IL-1R1^−/−^ mice (**Fig. 1.F**). Semi-quantitative analysis of the lung histology indicated a distinct increase of cell infiltration, mucus production and collagen deposition in d/d mice upon repeated LPS challenges as compared to untreated control mice. Numerous inflammation parameters were reduced in d/d x IL-1R1^−/−^ mice compared to d/d, but did not reach statistical significance likely due to the low number of surviving d/d mice (**Fig. 1.A** and **F-J**). In conclusion, the present data support the notion that in response to LPS challenge, IL-1β participates in inflammation and fibrosis in F508del CFTR mutants, and that genetic ablation of IL-1R1 signaling has a protective effect.

### 2. Increased inflammatory response after *P. aeruginosa* injection in cystic fibrosis lung

**Figure 2:**
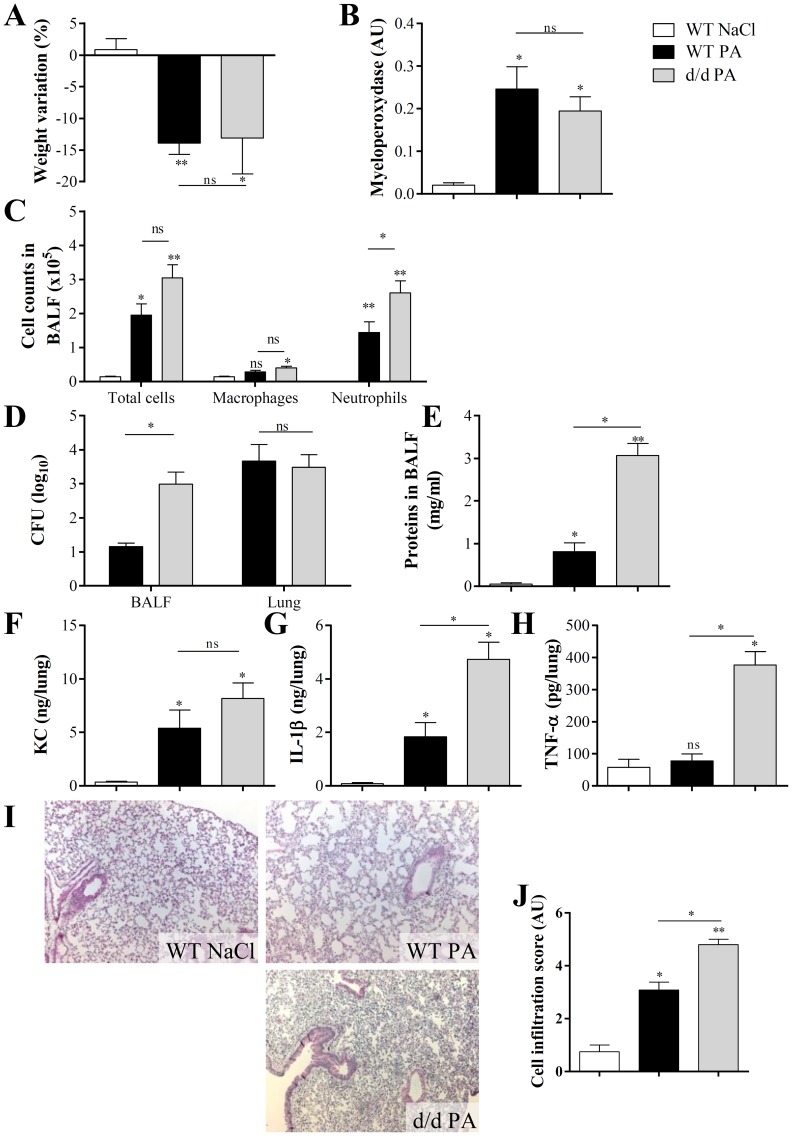
*P. aeruginosa* infection induces an increased inflammation in d/d mice. WT and d/d mice received 2×10^5^ CFU of *P. aeruginosa* PA011 intranasally, and WT mice treated with NaCl 0.9% were used as control. Body weight variation 20 h after infection is shown in (A). Myeloperoxidase activity was quantified (B) and absolute numbers of cells; (macrophages and neutrophils) were measured in BALF (C). Bacterial load (total CFU) was determined in BALF and in lung homogenate (D). Total protein concentration was evaluated in BALF (E) and KC (F), IL-1β (G) and TNF-α (H) were measured in lung homogenate. Cell infiltration was observed on H&E stained slide (I) and scored (J). (n = 5–6) Values are in mean +/− SEM; * for p<0.05; ** for p<0.01 and *** for p<0.001. ns for non-significant.

To further support this hypothesis in another model of CF pathology we used *P. aeruginosa* infection, a common lung infection occurring in CF patients. We established the model in our facility previously and showed that antibody neutralization of LPS inhibited acute and fatal *P. aeruginosa* induced pneumonia [30]. First, a *P. aeruginosa* (PA011) infection was performed in WT and d/d mice, WT mice administered saline were used as control. Twenty hours after *P. aeruginosa* infection, all mice developed severe pathology, as indicated by weight loss (**Fig. 2.A**). Lung inflammation, analyzed by the myeloperoxidase (MPO) activity, total cell and neutrophil counts in BALF was significantly increased in all groups compared with uninfected mice (**Fig. 2.B** and **C**). The bacterial load in the lung at 20 h did not differ between the groups (**Fig. 2.D**). However, bacterial load (CFU) detected in BALF of d/d mice was two logs higher in d/d mice than in WT mice (**Fig. 2.D**). Increased CFU in BALF of d/d mice was associated with heightened neutrophil recruitment as compared with WT mice (**Fig. 2.C**). This correlated with increased epithelial injury in d/d mice, compared to WT mice (**Fig. 2.E**) indicating that pulmonary epithelial damage and protein release were enhanced in d/d mice compared to WT mice (**Fig. 2.G** and **H**). KC chemokine (CXCL1), known to attract neutrophils is increased in all groups after *P. aeruginosa* infection, as compared with uninfected mice (**Fig. 2.F**). In d/d mice, IL-1β and TNF-α levels were substantially higher in the lung than in WT mice. Histological analysis showed augmented cell infiltration in d/d mice as compared with WT mice after *P. aeruginosa* infection (**Fig. 2.I** and **J**).

### 3. IL-1β antibody neutralization attenuates *P. aeruginosa*-induced inflammation

**Figure 3:**
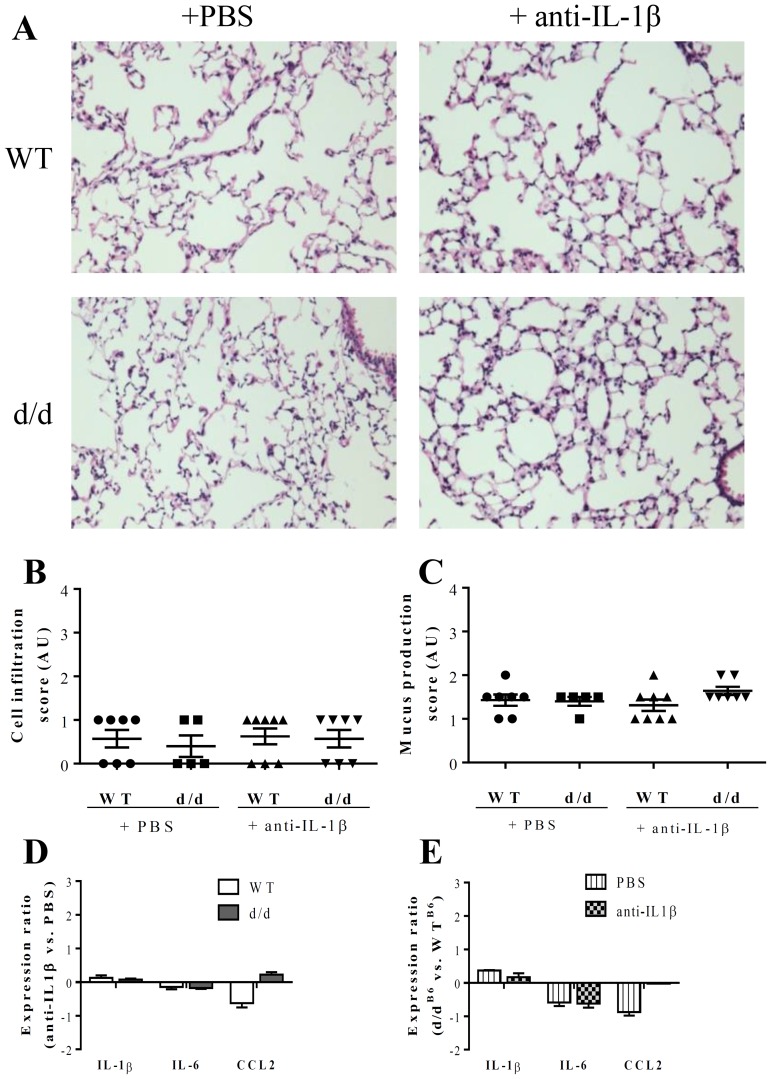
Anti-IL-1β antibody treatment has no significant effect on lung ultrastructure, nor on IL-1β, IL-6 and CCL2 mRNA production in lungs. Wild type (WT) and F508del CFTR mutation homozygote (d/d) mice (14-week-old females and males) were treated intra-peritoneally, once a week for 8 weeks, an anti-IL-1β antibody (10 mg/kg), or PBS. Mice were euthanatized 1 week after the last treatment and lung structure was observed on H&E stained slides observed at ×20 magnification (A). Cellular infiltration (B) was quantified on these H&E slides and mucus production (C) was analyzed on CAB stained lung slides. Lung injury score was recorded in anti-IL-1β antibody treated mice compared with PBS treated mice. (D and E) mRNA production of IL-1β, IL-6 and CCL2 was measured, and normalized with 2 housekeeping gene expression (*Hprt1* and *Gapdh*). The effect of anti-IL-1β antibody treatment compared to PBS control in WT and d/d animals is shown in (D), and the effect of F508del CFTR mutation compared to WT is shown in (E). (n = 6–7)

First, to study IL-1β function at steady state in the lung, we investigated the effect of a long-lasting anti-IL-1β antibody treatment in wild type mice or in mice with F508del CFTR mutation (d/d). Following 8-week antibody treatment, the young adult mice (14-week-old females and males) were investigated. The treatment was well tolerated; no mortality or morbidity was observed in any of the experimental groups. Histological analysis did not reveal any significant differences of cell recruitment, mucus production and collagen deposition in the lung (**Fig. 3.A**-**C**) between WT and d/d mice treated with control PBS or anti-IL-1 antibody. Lung mRNA expression of *Il1b*, *Il6* and *Ccl2* was assessed by qPCR and no significant difference was found among the control and the anti-IL-1β antibody administered groups (**Fig. 3.D** and **E**). Therefore, this investigation suggests that under basal conditions, in the absence of stimulation, no significant difference is detected between d/d and WT control mice. The F508del CFTR murine model (on C57BL/6 background), displayed no significant spontaneous lung inflammation under these conditions. Furthermore, long-term IL-1β antibody treatment had neither a significant effect on pulmonary morphology, nor on basal expression of *Il1b*, *Il6* and *Ccl2*, suggesting that IL-1β plays no significant role in steady state conditions.

**Figure 4:**
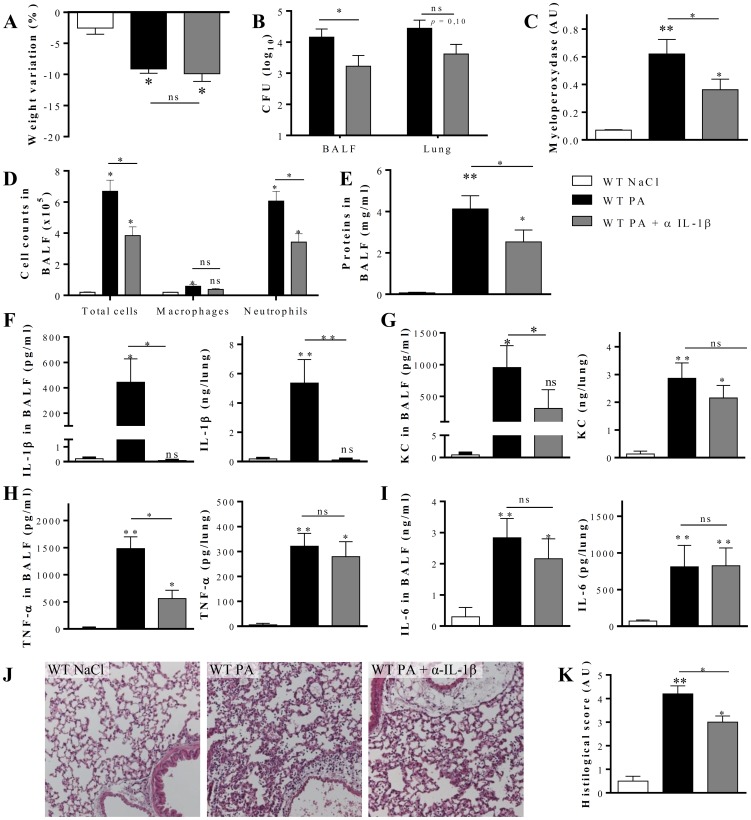
IL-1β participates in *P. aeruginosa*-induced inflammation at 20 h in WT mice. WT mice received 10^6^ CFU of *P. aeruginosa* PA011 intranasally, and WT mice treated with NaCl 0.9% were used as control. A group of WT mice was also treated intraperitoneally with anti-IL-1β antibody, 200 µg/mice, 15 h and 1 h before infection. Body weight variation 20 h after infection is shown in (A). Bacterial load was determined in BALF and in lung homogenate (B), myeloperoxidase activity was quantified (C) and absolute numbers of cells, (macrophages and neutrophils) were measured in BALF (D). Protein concentration was evaluated in BALF (E) and IL-1β (F), KC (G), TNF-α (H) and IL-6 (I), were measured in BALF and in lung homogenate. Cell infiltration was observed on H&E stained slide (J) and scored (K). (n = 5–6) Values are in mean +/− SEM; * for p<0.05; ** for p<0.01 and *** for p<0.001. ns for non-significant.

Subsequently, the role of IL-1β upon *P. aeruginosa* infection was studied using neutralizing antibodies in C57BL/6 mice. Mice were infected by intranasal instillation with 10^6^ CFU of *P. aeruginosa* (strain PA011) in order to induce an acute inflammation analyzed at 20 h. To test the role of IL-1β, mice received two peritoneal injections of 200 µg neutralizing antibodies 15 h and 1 h before infection. As reported before (Figure 2 and in [30]), infection of C57BL/6 mice with *P. aeruginosa* caused a rapid weight loss (**Fig. 4.A**), with a bacterial load of 10^4^ CFU in BALF and total lung (**Fig. 4.B**). *P. aeruginosa* infection provoked neutrophil recruitment (revealed by myeloperoxidase activity measurement and cell counts in BALF), and epithelial damage suggested by an increase of proteins in BALF in all groups post infection (**Fig. 4.C**-**E**). However, IL-1β neutralization had a distinct effect on infection and lung inflammation. Indeed, IL-1β blockade significantly reduced bacterial load in BALF, and to a lesser extent in lung (**Fig. 4.B**), suggesting a substantial difference in bacterial clearance between the two groups. This was correlated with a decrease in neutrophil recruitment (**Fig. 4.C** and **D**) and an inhibition of protein in BALF as measure of epithelial damage (**Fig. 4.E**). Moreover KC and TNF-α production in BALF were diminished (**Fig. 4.G** and **H**), whereas IL-6 was not modified (**Fig. 4.I**). In agreement with this, histological analysis showed reduced cell infiltration in anti-IL-1β treated mice as compared with WT untreated mice after *P. aeruginosa* infection (**Fig. 4.J** and **K**). These data suggest that IL-1β antibody neutralization may attenuate *P. aeruginosa*-induced acute lung inflammation.

## Discussion

CF patients display increased susceptibility to chronic infections with opportunistic bacteria, excessive lung inflammation and fibrosis leading to fatal loss of function eventually [6]. *P. aeruginosa* has been shown to induce IL-1β through NLRC4 inflammasome activation [9], [10]. CFTR deficient mice were found to be more susceptible to *P. aeruginosa* infection (acute [14], [15] and chronic [16]) and have an exacerbated inflammatory response to LPS and *P. aeruginosa*. Furthermore, F508del CFTR mutant alveolar macrophages display an enhanced IL-1β production in response to LPS stimulation [18]. Thus, we were interested in clarifying the role of IL-1β in the resolution of *P. aeruginosa* infection, in a murine model with the most common CF mutation F508del CFTR [21]–[23]. Here, we show that, after *P. aeruginosa* infection, excessive activation of IL-1β in F508del CFTR (d/d) mice compared to WT was accompanied by increased CFU in BALF, inflammation and lung damage. Further we show that a therapeutic antibody administration attenuates inflammatory response in an acute model of infection using WT mice.

In CF patients bacterial infections persist and their chronicity facilitates lung inflammation, and subsequent tissue remodeling with severe loss of function [1]. CF lungs display excessive immune response, with increased neutrophil recruitment [8]. We first investigated the role of IL-1β under pathologic conditions, induced by repeated *P. aeruginosa*-LPS challenges. We observed LPS-induced mortality in d/d mice, that was absent in d/d x IL-1R1^−/−^ double mutants, and a higher inflammation in d/d mice compared to the double mutant, suggesting a detrimental role of the IL-1R1 pathway in LPS-challenged d/d mice (Fig. 1). The deficiency in IL-1R1 apparently protected the mice against LPS-induced mortality in d/d mice. *P.aeruginosa*-LPS challenges were responsible for leucocyte recruitment, including neutrophils. Despite increased cell infiltration in the lung of d/d mice, collagen deposition and mucus production were only slightly increased after multiple LPS challenges. Repeated LPS challenges did not cause epithelial damage and chronic inflammation, which we explain by tolerance induction [31], that is the reason to switch to *P. aeruginosa* infection.

No spontaneous inflammation was found in young adult F508del CFTR mutant on C57BL/6 genetic background under our conditions. Whereas signs of inflammation and tissue remodeling develop early in most CF patients [32], results on basal inflammation in murine models are variable [23]. These data are consistent with published data on the absence of spontaneous pathology such as mucus plugging, neutrophil accumulation or bronchiectasis, in young mice deficient or mutated for the *Cftr* gene [15], [33]–[35], but neutrophilic inflammation has been reported in absence of infection [36], [37]. Spontaneous lung pathology development seems to be dependent on the age of mice [37], genetic background and animal facility health status [23]. However, in CF mutant mice challenged with pro-inflammatory agents (*P. aeruginosa*, LPS) enhanced injury and inflammation compared to wild type was reported in several independent studies [14], [15], [26], [38].

Therefore, to get closer to a model of lung infections that occur in CF patients, we used an acute *P. aeruginosa* infection model, which is the most current pathogen found in CF patient [7], in F508del CFTR mice. Twenty hours after *P. aeruginosa* infection, d/d mice displayed increased neutrophil recruitment in BALF as compared with WT mice (Fig. 2). They also showed an increase in bacterial load and cytokine production, such as IL-1β and TNF-α, in BALF. These results suggest a higher sensitivity to infection in d/d mice, consistent with previous studies in CFTR mutant mice, showing an augmentation of bacterial load and production of pro-inflammatory cytokines after *P. aeruginosa* infection [14], [15], [38]. The enhanced production of IL-1β compared to WT after infection was associated with increased epithelial damage, cell infiltration and CFU (Fig. 2). Then, we analyzed the effect of IL-1β neutralization at steady state in the lung. We found that in d/d as well as in WT mice, IL-1β antibody had no significant effect on survival. Furthermore d/d and WT mice displayed no difference in basal *Il1b* mRNA expression, and repeated anti-IL-1β antibody administration had no significant effect on basal inflammatory parameters and cytokine production. However, our data suggest that IL-1β may cause an excessive and pathologic inflammation in challenged CF mutant lungs. IL-1β over-expression in d/d mice infected with *P. aeruginosa* was accompanied by a higher bacterial load. Indeed, excessive production of inflammatory cytokines has been associated with bacterial persistence in other studies as well. High concentrations of cytokine, such as IL-1β, IL-6 or TNF-α, enhance intracellular and extracellular bacterial growth of *P. aeruginosa*, or *S. aureus* in the presence of monocytes [39], [40]. Furthermore, IL-1β release was linked to mortality in a murine model of *P. aeruginosa* infection after thermal injury [41].

Therefore, we tested the effect of a therapeutic antibody against murine IL-1β in our acute *P. aeruginosa* infection model in WT mice. IL-1β neutralization has been successfully tested previously with this antibody in a model of intestinal inflammation [25]. Twenty hours after *P. aeruginosa* infection, IL-1β antibody administration caused a decrease in the inflammatory parameters and in bacterial load, as compared with NaCl-treated mice. This is consistent with studies by Schultz *et al*. who showed that inhibition of the IL-1R1 pathway by either the IL-1R1 mutation or application of the IL-1β antagonist IL-1RA improved antibacterial host defense and reduced pro-inflammatory cytokine production [42]. Reduced neutrophil recruitment by IL-1β neutralization could be due to reduced endothelial activation, as proinflammatory cytokines activate endothelial cells. For instance, TNF-α and IL-17 synergize to induce in cultured endothelial cells the expression of P- and E-selectin, as well as neutrophil chemokines, increasing neutrophil transmigration [43]. IL-1β produced by activated monocytes was shown to augment the expression of adhesion molecules (ICAM-1, E-selectin) *in vitro* [44]. Thus, the diminished leucocyte recruitment we observe with IL-1β neutralization could be related to a reduced endothelial activation. Abnormal recruitment and metabolic adaptation of neutrophils in human CF airways has been demonstrated [45], the molecular mechanism remains to be established but likely involves IL-1β signaling based on the arguments mentioned above. We cannot exclude a role of lung fibroblasts and myoblasts. Indeed, IL-1β stimulated human cardiac fibroblasts overexpress adhesion molecules and neutrophil chemoattractant [46].

In conclusion we propose that the IL-1β pathway is critical to drive excessive and detrimental inflammation in F508del mouse model of CF. We show here that antibody neutralization of IL-1β is well tolerated in mice, has no effect on the unchallenged lung in WT or F508del CFTR mice, whereas it can reduce pathology induced by acute bacterial lung infection.
